# Gait variability development in autistic individuals across childhood and adulthood

**DOI:** 10.21203/rs.3.rs-9622252/v1

**Published:** 2026-05-07

**Authors:** Robin L. Shafer, Jingying Wang, Hang Qu, Joelle P. Simpson, Matthew Terza, Desirae J. Shirley, Walker S. McKinney, Stormi Pulver, Ann-Marie Orlando, Regilda A. Romero, Bikram Karmakar, Matthew W. Mosconi, Zheng Wang

**Affiliations:** University of Kansas; University of Florida; University of Florida; University of Florida; University of Florida; University of Florida; University of Kansas; Emory University; University of Florida; University of Florida; University of Wisconsin-Madison; University of Kansas; University of Florida

**Keywords:** Unconstrained walking, canonical correlation analysis, gait variability, composite score, sensorimotor development, autism spectrum disorder

## Abstract

Atypical sensorimotor behaviors are prevalent in autism spectrum disorder (ASD), often emerging in infancy and persisting into early adulthood. Prior focus on qualitative metrics and developmentally specific motor skills has limited understanding of sensorimotor differences in ASD across the lifespan. The present study quantified gait kinematics in ASD across a wide age range, including analysis of 60 autistic individuals (ages 4–35 years) and 53 age-, sex-, and performance IQ-matched neurotypical (NT) controls. Autistic individuals differed from NT controls on seven gait variables reflecting within-subject variability of gait kinematics. These seven gait variability metrics were integrated into a canonical correlation analysis (CCA) with select demographic features (group, age, sex, IQ) to derive a composite gait variability score that accounts for the multicollinearity of gait variability and demographic metrics. The composite score revealed increased gait variability in autistic individuals relative to NT controls. Gait variability was negatively associated with age across the sample. Age-by-group interactions suggested that gait variability is more severely elevated in autistic individuals during adolescence and adulthood relative to childhood. Increased gait variability also was associated with more severe clinically rated ritualistic behaviors in autistic individuals. This study leverages a novel approach to define a multidimensional component score of gait variability in ASD from childhood to adulthood. Results indicate that gait variability is elevated in ASD, and that the severity of variability differences increases with age during adolescence and into adulthood. These findings suggest that autistic individuals show an attenuated development of sensorimotor feedback and motor planning processes that are involved in maintaining accuracy and stability of movements.

## ABBREVIATED SUMMARY

This study used multiple gait variability metrics and demographic factors to create a gait variability (instability) composite score for characterizing autistic and neurotypical gait development. We found that gait variability decreases with age in autistic and neurtypical individuals, but autistic individuals show slower change and persistently elevated gait variability.

## INTRODUCTION

Autism spectrum disorder (ASD) is a neurodevelopmental condition characterized by social-communication challenges, restricted interests and stereotypic behaviors^1^. Sensorimotor impairments are highly prevalent in ASD, affecting 35–87% of autistic individuals^2–5^. Sensorimotor differences are also closely associated with more severe autistics traits, poorer long-term outcomes, and reduced quality of life ^6–9^. They commonly manifest early in infancy and persist into adulthood, impacting both fine and gross motor systems^10–15^. Most previous studies of sensorimotor impairments in ASD have relied on clinical assessments and ratings of motor behaviors that broadly categorize motor skills (e.g., fine and gross motor)^2,10,16,17^ or on quantitative, objective measures that are constrained to developmentally specific actions (e.g., spoon feeding, handwriting)^18,19^. While clinical assessments offer valuable diagnostic insights, their broad scope preclude a mechanistic understanding of sensorimotor differences in ASD. Studies of developmentally constrained behaviors also are limited in their ability to clarify how sensorimotor differences vary across the lifespan in ASD.

Measurement of movement features during natural walking (i.e., gait) provides an important framework for understanding sensorimotor behavior and development^20^. As a fundamental gross motor skill, gait supports mobility and enables individuals to explore and interact with their environment in ways that diversify sensorimotor, cognitive, and social experiences^21–23^. Gait also undergoes a prolonged and complex maturational time course extending from the first year of life through the third decade^24–28^. Typical gait development is characterized by rapid maturation of spatiotemporal and joint kinematic patterns in the first few years of walking (2–5 years of age) including transitioning to striking with heel first (e.g., instead of flat foot), introduction of reciprocating arm swing, and reduced coactivation of agonist-antagonist muscles^25^. Stride length increases across childhood into adolescence, while gait speed increases primarily during adolescence and early adulthood^24,26^. Variability of stride length decreases rapidly in early childhood, reaching adult levels by mid-adolescence^24,27^. Similarly, cadence, stride time and stride duration variability decrease rapidly throughout childhood, with a slower, steadier decrease during adolescence as these metrics approach adult levels^24,26–28^. These developmental changes are present even when controlling for differences in body size (e.g., height)^24,27^. Aging-related behavioral changes in gait also serve as critical and sensitive biomarkers for multiple health-related outcomes, including independence during aging and in clinical conditions, adaptive functioning, and well-being^29–32^.

Findings on spatiotemporal and joint kinematics during gait in ASD have been mixed. For example, several studies have found decreased step and stride length^33–35^, reduced cadence ^33,36^ and velocity^33,36^, as well as increased double support time (i.e., time when both feet are in contact with the floor or other support surface)^33^ in autistic compared to neurotypical (NT) individuals. In contrast, other studies have reported opposite patterns^37,38^ or no significant between-group differences in these metrics ^14,34,35,38^. Similarly, while some studies have noted differences in joint angles during gait, the specific joints for which differences are reported vary across studies^34,38,39^. Together, inconsistencies in both metric selection and reported outcomes limit our ability to identify gait profiles associated with ASD or to characterize how gait differences in ASD may vary across the lifespan^34,38,39^.

Variability of findings on spatiotemporal and joint kinematic differences in ASD may reflect inherent heterogeneity of sensorimotor behavior among autistic individuals^33,39^ that covaries with key demographic features, including age, sex, IQ, or other unidentified characteristics (e.g., etiology)^40,41^. In contrast to findings from studies examining mean values for select gait features, findings from studies analyzing variability (i.e., standard deviation) of gait metrics – both within a movement and across repeated movements – appear to show more consistent differences between autism and NT controls. Multiple studies have identified elevated gait variability in autistic children (ages 4–18 years) across several metrics, including stride length, stride width, stride time, velocity, and foot-shank coupling (i.e., ankle) angle^14,37,42^. In other conditions, such as aging and neurodegeneration, gait variability metrics have proven more sensitive for detecting atypical gait patterns than mean values of spatiotemporal metrics^43^. Moreover, changes in variability of gait dimensions with age reflect neural processes distinct from those underlying spatiotemporal metrics^43^, suggesting that studying gait variability may provide unique insights into neurodevelopmental mechanisms associated with ASD. Nonetheless, inconsistencies remain regarding which gait variability metrics are selected for analysis, which metrics are most robust to differences between autistic and NT individuals, and the extent to which variability relates to demographic factors (e.g., age, sex, IQ). These gaps underscore the need for approaches that integrate gait variability across spatiotemporal and joint kinematic metrics and compare them with demographic features to more comprehensively characterize gait development from childhood through adulthood in autistic individuals.

The objectives of this study were to (1) optimize measurement of gait variability differences in ASD by deriving a composite gait variability metric that accounts for multicollinearity across individual gait metrics and demographic characteristics, and (2) characterize age-related changes (4–35 years) in gait variability in autistic and NT individuals. For the first objective, we applied a multi-dimensional analytic approach, canonical correlation analysis (CCA), to derive a composite gait variability score (CGVS) for each participant. The CCA model reflects relationships both between and within two multivariate datasets – gait variability (dataset 1) and demographic variables (dataset 2) – while accounting for multicollinearity among measures^44–46^. Consistent with prior literature, we hypothesized that autistic individuals would show elevated gait variability^14,37,42^, measured with the CGVS, relative to NT individuals, and that CGVS would decline with increasing age in both groups^14,24,43,47–49^. The literature is mixed on whether motor variability in autistic individuals converges with or diverges from NT individuals with increasing age^14,15,50,51^, so we did not have *a priori* hypotheses about the group-by-age interaction. Finally, to determine whether gait variability relates to select traits associated with ASD, we explored the relationship between CGVS and clinical autistic traits.

## METHODS

Participants were examined at one of three sites: the University of Texas Southwestern Medical Center (UTSW), the University of Kansas (KU), or the University of Florida (UF). Diagnostic standards, testing procedures, and task instructions were consistent across sites. The study protocol was approved by the Institutional Review Boards (IRB) at UTSW and Children’s Hospital of Dallas (STU052013–4), KU Medical Center (STUDY00140269), and UF (201801378). Participants 18 years of age or older provided written consent. Minors or adults under legal guardianship provided assent in addition to guardians’ written consent based on the Declaration of Helsinki.

### Participants

Sixty autistic individuals ages 4–35 years and 53 NT controls matched on age, sex, anthropometry and performance IQ participated in this study. Table 1 shows the demographic and clinical characteristics of autistic individuals and NT controls (see Supplementary Table 1 for demographic and clinical feature breakdowns for UTSW, KU, and UF cohorts separately). Autistic individuals were recruited through community advertisements, clinical programs, institutional research registries, and SPARK Research Match (https://www.sfari.org/resource/research-match/). Control participants were recruited through flyers and word of mouth. The UTSW cohort consisted of 23 autistic individuals and 23 NT controls; the KU cohort included 32 autistic individuals and 23 NT controls; the UF cohort consisted of 5 autistic adults and 7 NT controls. All study procedures were approved by the Internal Review Boards at UTSW, KU, and UF, and written informed consent was obtained from all participants (or their legal guardians) according to the Declaration of Helsinki.

ASD diagnosis was established following the Diagnostic and Statistical Manual of Mental Disorders, 5^th^ Edition (DSM-5)^52^. Autistic individuals at UTSW and KU screened ≥ 15 on the Social Communication Questionnaire (SCQ)^53^, and participants in the UF cohort scored ≥ 32 on the Autism Spectrum Quotient (AQ-50) for adults^54^ and ≥ 65 on the Social Responsiveness Scale-2 (SRS-2) adult self-report^55,56^. Eligible individuals were invited for a diagnostic evaluation and completed the Autism Diagnostic Observation Schedule, 2nd Edition (ADOS-2)^57^ administered by a research-reliable study clinician. The UTSW and KU sites additionally administered the Autism Diagnostic Interview–Revised (ADI-R)^58^. In addition to standardized measures, expert clinical opinion was used to confirm ASD diagnosis at all sites. Across sites, all autistic individuals were required to have a full-scale IQ (fs-IQ) ≥ 70 to participate in this study. No autistic individuals had any known genetic or metabolic disorder associated with ASD (e.g., Fragile X syndrome, Tuberous Sclerosis, Phelan McDermid syndrome, Burnside-Butler syndrome) based on medical history reviews.

NT controls were enrolled if they scored < 8 on the SCQ during initial screening at UTSW or KU. NT controls enrolled at UF had a score of < 22 on the AQ-50 or a score of < 60 on the SRS-2. Across sites, NT controls were excluded if they (a) had any major psychiatric (e.g., schizophrenia, bipolar affective disorder, and obsessive compulsive disorder) or neurological (e.g., epilepsy, cerebral palsy, spinal cord injury, and ataxia) disorders prior to or at the time of study participation; (b) had a family history of ASD or other forms of neurodevelopmental disorders in first- or -second-degree relatives; (c) had a family history of any major psychiatric disorders in first-degree relatives, or (d) had a fs-IQ < 80.

No participant (autistic or NT control) had a history of birth asphyxia or non-febrile seizure. One autistic adult at UF reported a prior history of substance abuse. Thirteen UTSW (8 on antidepressants, 7 on stimulants, 1 on sedatives), six KU (4 on antidepressants and 2 on stimulants) and four UF (1 on antipsychotics, 3 on antidepressants, 2 on antihypertensives) participants reported being on medication within 48 hours of gait testing. All participants were able to ambulate independently without assistive devices. All participants completed the Repetitive Behavior Scale-Revised (RBS-R)^59^ and had their IQ assessed using the Wechsler Abbreviated Scales of Intelligence, 2nd Edition (WASI-II)^60^.

### Apparatus and Procedures

The apparatus and procedures were consistent across sites. Prior to testing, 16 passive reflective markers were attached to participants’ major joints based on the industry-standardized Plug-in-Gait Lower Body template (Vicon, Centennial, Co). Kinematic data were recorded using Nexus 3D motion capture systems. The motion capture systems at UTSW and KU included 10 Vicon Bonita 10 near-infrared cameras with a spatial error of 0.5 mm and a sampling rate of 250 Hz. The motion capture system at UF incorporated 12 VICON Vantage 5 cameras and a spatial error of 0.2 mm and sampling rate of 120 Hz. The walking distance was 5 meters at UTSW and KU and 7 meters for UF.

Participants started each trial by standing upright with their feet shoulder width apart and arms by their sides. After receiving a verbal cue, participants began walking continuously at their self-selected speed toward the finish line. Each individual completed 3–5 trials. Practice trials were administered prior to data acquisition to ensure participants felt comfortable wearing reflective markers and understood task instructions. To minimize variability in the rates at which individuals increased or decreased their walking speed at the beginning and end of each trial, only kinematic data representing individuals’ “preferred speed” were selected and post-processed. Heel strikes and toe-offs of each leg were manually labeled on the raw kinematic data by trained scorers and cross-checked among scorers (JW, JPS, DJS, WSM, and ZW). Kinematic data were then exported, filtered, and analyzed using custom scripts in MATLAB 2023a (MathWorks, Inc., Natick, MA). A low-pass, zero-lag, 4th -order Butterworth filter was applied at a cutoff frequency of 7 Hz to filter the raw kinematic data.

Spatiotemporal variables of gait consisted of the step width (i.e., mediolateral distance between heel markers of two consecutive heel strikes), step velocity (i.e., ratio of step length and step time), and cadence (i.e., number of steps per minute). Kinematic features of gait included relative angles of the ipsilateral hip (i.e., pelvis-thigh), knee (i.e., thigh-shank), and ankle (i.e., shank-foot) joints at heel-strikes and toe-offs within the sagittal plane. Within-individual standard deviation was calculated for each gait variable across all walking trials. These nine gait standard deviation measures were used as candidate variables to derive the composite gait variability score (CGVS).

### Clinical Assessments

To test relationships between gait and clinical behaviors, associations with ADOS-2^57^ and RBS-R^59^ were examined for the autistic participants. The ADOS-2 is a semi-structured assessment of play, social abilities, communicative skills, and imaginative use of materials. ADOS-2 calibrated severity scores were calculated to allow comparisons across different modules and age ranges ^61,62^. Higher scores reflect increased clinical severity of ASD. The RBS-R is a parent/self-report measure of the presence and severity of restricted, repetitive, and stereotyped behaviors. It contains six subscales, including stereotyped behavior, self-injurious behavior, compulsive behavior, ritualistic behavior, sameness behavior, and restricted behavior. Both subscale and total scores were calculated for each individual. Higher scores reflect more severe repetitive behaviors in autistic individuals.

### Statistical Analyses

#### Demographic and clinical characteristics.

Demographic (age, sex, fs-IQ, verbal IQ, and performance IQ), anthropomorphic (height and weight), and clinical characteristics (ADOS-2 calibrated severity scores and RBS-R total scores) were compared between autistic individuals and NT controls using independent-sample t-tests or Chi-square tests, as appropriate.

#### Composite gait variability score (CGVS).

Two procedural steps were implemented to derive the CGVS for each individual. *Step 1* involved identifying gait standard deviation measures that significantly differentiated autistic individuals from NT controls. To this end, a multivariate analysis of covariance (MANCOVA) was conducted with group (ASD vs. NT) as a fixed factor, nine gait standard deviation measures as dependent variables, and height and weight as covariates. Height and weight were included in this model because anthropometry significantly affects gait characteristics, especially for studies with a broad developmental age range^63–65^. False discovery rate (FDR) correction for multiple comparisons was applied to control Type *I* error, and statistical significance was interpreted at *p*_FDR_ < 0.05^66^.

**Figure 1:**
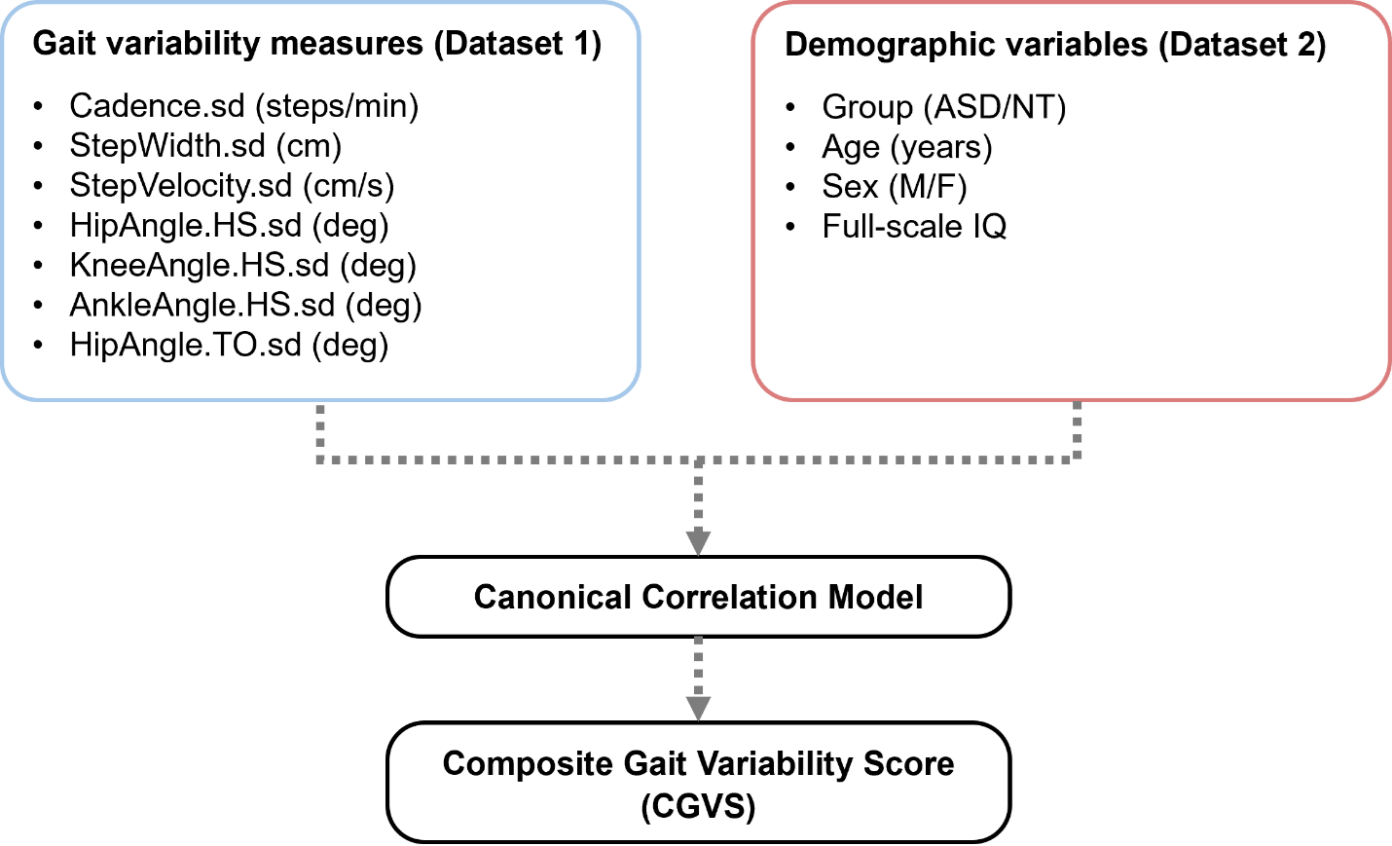
Derivation of CGVS using CCA. Dataset 1 consisted of seven gait standard deviation measures that significantly differentiated autistic individuals from NT controls (Table 2). Dataset 2 included four demographic variables critically affecting gait variability in autistic individuals and NT controls. sd = standard deviation; HS = heel strike; TO = toe-off.

Canonical correlation analysis (CCA) was implemented as *Step 2* using two datasets (Fig. 1). Dataset 1 included seven gait standard deviation measures determined to significantly differentiate autistic from NT individuals in the *Step 1 analysis*, and Dataset 2 consisted of four demographic variables including group, age, sex, and fs-IQ. Four canonical variates were derived from the canonical correlation model because the smaller dataset (i.e., Dataset 2) consisted of four variables. Canonical variates were calculated following the formula:

$$\rho_{i}=\frac{\text{cov}(U_{i},V_{i})}{\sigma (U_{i})\sigma (V_{i})}$$

where ${\text{ρ}}_{\text{i}}$ represents the i^th^ canonical variate, $\text{cov}(U_{i},V_{i})$ represents the covariance matrix of the Datasets 1 and 2, and $\sigma (U_{i})$ *and* $\sigma (V_{i})$ stand for the standard deviation matrix of each individual dataset. Canonical variates were interpreted as significant when *p*_raw_ < 0.05. CGVS was then derived based on the statistically significant canonical variate(s) following the formula:

CGVS(i)=Cadence.coeff(i)×Cadence.sd+StepWidth.coeff(i)×StepWidth.sd+StepVelocity.coeff(i)×StepVelocity.sd+HipAngle.HS.coeff(i)×HipAngle.HS.sd+KneeAngle.HS.coeff(i)×KneeAngle.HS.sd+AnkleAngle.HS.coeff(i)×AnkleAngle.HS.sd+HipAngle.TO.coeff(i)×HipAngle.TO.sd.(3)

where CGVS (i) represents the i^th^ canonical gait variability score whose canonical variate was statistically significant; coeff (i) stands for the unstandardized canonical coefficient of the i^th^ linear combination of gait standard deviation measures included in Dataset 1 (U_*i*_), sd stands for standard deviation, HS stands for heel strikes, and TO is toe-offs. Unstandardized canonical coefficients (i.e., weightings) represent the degree to which changes to one gait standard deviation measure in Dataset 1 correlate with changes in the corresponding canonical variate of Dataset 2, when all remaining gait standard deviation measures are held constant. Only the first canonical variate ($\rho_{1}$) was statistically significant (see Results), so only the first CGVS was derived for each individual.

#### Diagnostic sensitivity and specificity of CGVS relative to discrete gait standard deviation measures.

Receiver Operating Characteristic (ROC) curve analysis^67^ was performed as a *post hoc* analysis to examine the specificity and sensitivity of CGVS against discrete gait standard deviation measures for differentiating autistic and NT individuals. The area under the curve (AUC) was derived to identify the outcome measure with optimal specificity and sensitivity.

#### Demographic and clinical correlations.

Spearman’s rank-order correlation analyses were conducted to examine the relationship between CGVS and demographic variables in both groups and clinical ratings for autistic individuals, including age, fs-IQ, ADOS-2 calibrated severity score, and RBS-R subscale scores. The RBS-R self-injury subscale was omitted from these analyses due to restricted score variability in the sample. Correlations involving age, fs-IQ, and ADOS-2 scores were interpreted as significant at *p*_raw_ < 0.05. To account for multiple comparisons across RBS-R subscales, FDR correction was applied^66^ and correlations involving RBS-R subscale scores were interpreted as significant at *p*_FDR_ < 0.05.

#### Comparison of correlation coefficients between groups.

Fisher’s *z* transformation^68,69^ was applied to statistically significant correlations to test whether the strength of correlations between CGVS and demographic measures differed significantly between the ASD and NT groups.

## RESULTS

### Composite gait variability score (CGVS).

Table 2 summarizes MANCOVA results for gait variability comparisons across autistic individuals and NT controls (*Step 1* of deriving the CGVS). Seven of the nine gait variability measures that were analyzed significantly differentiated autistic from NT individuals after FDR correction. These seven measures included the standard deviations of cadence (Cadence.sd), step width (StepWidth.sd), step velocity (StepVelocity.sd), hip angle at heel strikes (HipAngle.HS.sd) and toe-offs (HipAngle.TO.sd), and knee (KneeAngle.HS.sd) and ankle angles at heel strikes (AnkleAngle.HS.sd). These gait variability measures comprised Dataset 1 of the canonical correlation model (*Step 2* of deriving the CGVS).

Only the first canonical variate $\left(\rho_{1}\right)$ was significant (Table 3), so it was used to derive the CGVS for each individual using the formula below:

CGVS=(0.048)×Cadence.sd+(0.240)×StepWidth.sd+(0.036)×StepVelocity.sd+(0.296)×HipAngle.HS.sd+(0.147)×KneeAngle.HS.sd+(0.210)×AnkleAngle.HS.sd+(0.096)×HipAngle.TO.sd(1)

The derived CGVS represents individuals’ overall gait variability during natural walking, accounting for the multicollinearity of each individual gait variability measure included in Dataset 1 and the demographic variables included in Dataset 2. All unstandardized canonical coefficients were positive (1) suggesting a positive relationship between each gait standard deviation measure and CGVS. As such, higher CGVS scores indicated greater overall gait variability.

**Figure 2:**
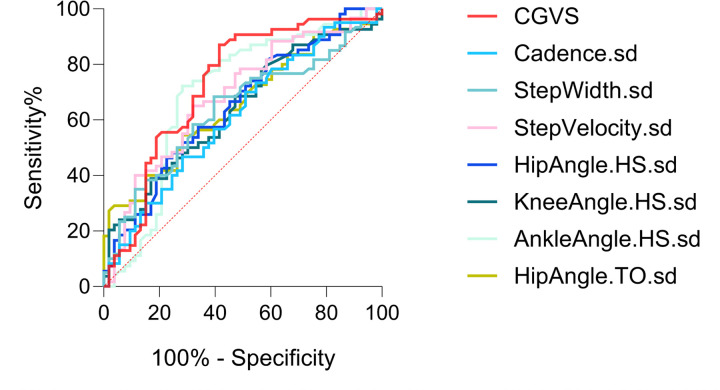
Diagnostic sensitivity and specificity of the CGVS and discrete gait metrics. Receiver operating characteristic (ROC) curves for the CGVS and each discrete gait metric. CGVS = composite gait variability score; sd = standard deviation; HS = heel strike; TO = toe-off.

ROC analysis demonstrated that CGVS achieved fair discriminative ability in differentiating autistic from NT individuals (Fig. 2 and Table 4). CGVS yielded the greatest area under the curve (AUC) value among the tested measures (AUC_CGVS_ = 0.729; 95% CI: 0.629–0.828).

### Demographic and clinical correlations.

**Figure 3:**
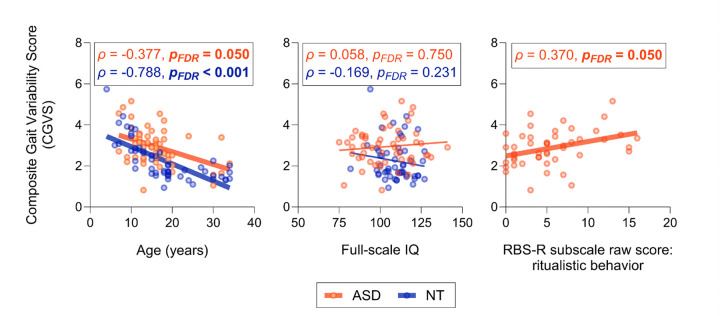
Clinical and demographic correlations with CGVS. Correlation analysis shows the relationship of CGVS with age (A), fs-IQ (B), and ritualistic behavior subscale score of RBS-R (C). Shaded areas show 95% confidence intervals of corresponding measurements for each group.

Lower CGVS was associated with increased age for both autistic individuals (ρ = −0.377, *p* = 0.005) and NT controls (ρ = −0.788, *p* < 0.001, Fig. 3A). Fisher’s *z* transformation revealed that the relationship between CGVS and age differed significantly between groups (*z* = −3.364, *p*_FDR_ = 0.001). Specifically, NT individuals showed a strong negative correlation between CGVS and age, whereas autistic individuals demonstrated a weaker negative correlation.

The CGVS was not associated with fs-IQ for autistic individuals (ρ = 0.058, *p* = 0.675) or NT control participants (ρ = − 0.169, *p* = 0.231; Fig. 3B). The relationship between CGVS and fs- IQ also did not differ significantly between groups (*z* = −1.143, *p*_FDR_ = 0.253).

Increased CGVS was associated with increased RBS-R ritualistic behavior subscale scores in autistic individuals (ρ = 0.370, *p*_FDR_ = 0.050; Fig. 3C). No other clinical associations with CGVS were identified (Supplementary Table 2).

## DISCUSSION

The present study used canonical correlation analyses to quantify gait variability differences in ASD. This approach allowed us to derive a composite metric of gait variability (CGVS) that accounts for the multicollinearity of spatiotemporal and joint kinematic variables, as well as demographic characteristics, and it provides a singular, powerful metric for comparing gait profiles in autistic and NT individuals from childhood through adulthood. Three key findings are reported: (1) autistic individuals show increased gait variability compared to chronological-age and sex-matched NT peers, suggesting that sensorimotor processes involved in modulating the variability of motor behavior are disrupted in autistic individuals; (2) the multidimensional CGVS derived here provided greater sensitivity to gait differences in ASD than any individual measurement of gait, suggesting it may offer important advantages for tracking gait development in ASD and identifying clinical and neurobiological correlates, and (3) gait variability, as represented by the CGVS, shows age-related reductions across childhood and into adulthood in autistic and NT individuals, but this age-related reduction is attenuated in autistic individuals. We also document that gait differences in ASD are independent of general cognitive abilities, indicating that sensorimotor differences in ASD occur across individuals with a range of developmental abilities within the non-intellecual disability range.

### Composite gait variability for understanding sensorimotor mechanisms of ASD.

Our findings demonstrate that variability of gait can be represented in a single composite gait variability score (CGVS) that distinguishes gait patterns in ASD from those in NT individuals. Specifically, autistic individuals showed higher CGVS than NT individuals, indicating greater variability of multiple gait features, consistent with prior findings^14,37,42^. We also found that mean spatiotemporal and joint angle metrics of gait did not differentiate autistic and NT individuals (Supplementary Table 3), which could indicate that autistic individuals show specific increases in gait variability that may be critical for distinguishing sensorimotor behaviors across autistic and NT individuals. The lack of significant group differences on mean gait measures could also be attributed to nonlinear age-associated changes that are documented in several mean gait metrics^24,48^, such that differences between autistic and NT gait may only appear at certain points in development, and between group effects may not be detectable if averaged across samples with broad age ranges. These results reinforce findings showing that elevated gait variability in autistic individuals relative to NT controls^14,37,42^ is more consistent across studies and ages than differences in mean spatiotemporal^33–38^ or joint kinematic measures^34,38,39^. Measures of gait variability are often more robust to changes in gait control (e.g., during development, aging, or disease) than mean spatiotemporal or kinematic measures^70,71^. Variability can indicate differences in stability and consistency of gait control even when averaged parameters (e.g., cadence or step length) show no or smaller differences.

The key mechanisms contributing to increases in gait variability in ASD are not yet fully elucidated, but consideration of gait variability differences in separate clinical conditions may provide important insights. Elevated gait variability is a characteristic feature of cerebellar ataxia^72^, and it is common in other populations that show motor or cognitive issues including Parkinson’s disease^43,72,73^, peripheral neuropathy^74^, and healthy aging ^75,76^. In these populations, elevated gait variability is associated with deficits in feedforward control of intralimb movements^72^, disrupted sensorimotor feedback processing^73,77^, and reduced cognitive and attentional capacity^73,76^, suggesting that each of these processes or their combination may contribute to elevated gait variability in autistic individuals. This hypothesis is consistent with findings of elevated motor variability across multiple motor behaviors and effector systems in autistic individuals including visually guided saccades^78,79^, precision grip force control^13,80–82^, reaching^83^, and postural control^84,85^, which implicate atypical feedforward processing, sensorimotor feedback processing, and cognitive motor control processing. During studies of gait and postural control, autistic individuals show a greater increase in gait and postural variability than NT individuals under sensory conditions that put greater demand on sensory feedback processing (e.g., walking or standing on a pliable surface), while increasing cognitive demand only increases variability of gait and postural control in NT individuals but not in autistic individuals, suggesting that atypical integration of sensory feedback may contribute more to elevated gait variability in autism than cognitive motor control processes^86,87^.

The CGVS described here showed greater than 80% sensitivity for detecting autism with specificity of up to around 60%, suggesting that multidimensional measurements of gait variability may provide unique power for tracking sensorimotor differences in ASD and establishing clinical and neurobiological correlates. Prior studies have assessed gait variability using only one or a small number of discrete metrics (e.g., standard deviation of stride length, time, or velocity), and results have been somewhat inconsistent in terms of which metrics appear to be most robust for differentiating autistic and NT individuals^14,37,42,88^, limiting the understanding of gait and broader sensorimotor differences in ASD. In contrast, the CGVS derived in the present study considers several discrete gait variability metrics, as well as demographic factors. This approach is particularly important for understanding sensorimotor differences across a clinically and neurobiologically heterogeneous condition such as ASD.

### Age-associated gait variability differences in ASD.

In our sample of individuals aged 4–35 years old, we observed a decrease in gait variability with increased age in both autistic and NT individuals. However, NT individuals showed a stronger age-related reduction in gait variability than autistic individuals suggesting a slower (or otherwise atypical) development of gait stability in ASD. This atypical course of motor development appears to lead to persistent elevations in gait variability through childhood that become more pronounced in later adolescence and adulthood. These results suggest that neurotypical processes supporting reductions of movement variability during gait in adolescence and adulthood are either attenuated or deviant in ASD, contributing to persistent elevations in gait variability through childhood and into adulthood. This finding contrasts with a prior study of gait variability across ages (4–16 years) in ASD^14^, which found that elevated gait variability in ASD is most pronounced at younger ages and normalizes during later childhood and adolescence. These discrepant findings could be attributed to our use of the CGVS, which integrates multiple discrete measures of gait variability, while the prior study assessed a smaller number (N = 3) of discrete gait variability measures. Distinct gait variability metrics may follow separate developmental trajectories which could lead to inconsistent age-associations when comparing metrics, as has been observed in normative development. Specifically, in normative development, temporal gait variability metrics (e.g., stride time variability) decrease dramatically from the onset of walking until around 6–8 years of age^24,43,47–49^, but spatial variability metrics (e.g., variability of stride width or knee angle) show little to no change in early childhood^48,49^. However, since the CGVS integrates multiple discrete gait metrics, it provides a more comprehensive quantification of gait variability that may be more sensitive to age- or diagnosis-related differences than individual measures. Differences across studies also could reflect heterogeneity within the autistic population both in terms of the extent to which gait variability is elevated and the rate at which gate variability shows age-dependent changes within individuals. The prior study of age-related changes in gait variability in ASD specifically assessed autistic individuals with ‘infantile’ or ‘atypical’ autism, including individuals with known genetic syndromes associated with autism, and excluding individuals with a diagnosis of Asperger’s syndrome or pervasive developmental disorder – not otherwise specified^14^. This suggests that their autistic sample had more severe autistic traits than the present study, which did not include individuals with known genetic syndromes or fs-IQ below 70 but did include individuals with milder autistic traits, some of whom likely would have met DSM-IV criteria for Asperger’s or pervasive developmental disorder – not otherwise specified.

Studies of age-associated changes (4–29 years^50^ and 10–20 years^51^) in precision grip force variability in autistic individuals have shown greater decreases in variability with increased age in ASD relative to NT, such that differences were most pronounced at younger ages, consistent with Manicolo et al.’s observations of gait variability in ASD. Other studies of trial-to-trial variability in saccadic eye-movement gain (ages 5–29 years)^50^ and postural control (ages 8–20 years)^15^ suggest that motor variability shows similar age-related reductions in autistic and NT individuals. Inconsistencies in findings of age-associated changes in motor variability in ASD highlight the need for future studies to examine gait variability at younger ages and in longitudinal samples to determine when gait variability differences first emerge and how they evolve across different individuals over time.

### Increased gait variability in autism implicates select brain networks.

Variability and mean gait parameters appear to be controlled by separate, but overlapping neurophysiologic mechanisms^73,89–91^, suggesting that our finding showing specificity of gait variability in differentiating autistic individuals from NT individuals may be associated with atypical function of select motor control networks in ASD. Neuropathology, brain stimulation, and neuroimaging studies indicate that gait variability is associated with cerebellar-cortical^72,73,91,92^ and cortico-cortical^73,89,93–95^ brain networks involved in motor planning, sensorimotor feedback processing, and coordination. Elevated variability of gait is associated with pathology in the cerebellum including cerebellar lesions and atrophy^72,92^, as well as reduced metabolism^73^. Increases in gait variability can also be induced by inhibiting activity in the cerebellum or posterior parietal cortex using transcranial magnetic stimulation^91^. These findings are consistent with cerebellar involvement in somatosensory and vestibular integration, coordination across limbs, and postural control, as well as the role of posterior parietal cortex in integrating multimodal sensory feedback for correcting motor error. Additionally, elevated gait variability in healthy and pathologic aging is associated with reduced involvement of the dorsal attention network, increased involvement of the default mode network when measured at rest^89,93^ and atypical functional connectivity within frontal-parietal networks^94,95^, consistent with involvement of cognitive and attentional control in attenuating inherent variability of movement.

Each of these brain networks has been implicated in ASD. Task-free neuroimaging studies have reported that, relative to NT controls, autistic individuals show increased functional connectivity between cerebellum and posterior parietal cortex, as well as primary sensory and motor cortices^96–98^, suggesting heightened intrinsic connectivity within cerebellar-parietal networks that integrate and translate sensory feedback information into corrective motor commands. In contrast, autistic individuals show reduced functional connectivity between cerebellum and dorsolateral prefrontal cortex^98^, suggesting reduced connectivity within frontal-cerebellar networks that are involved in motor planning. During tests of visually guided precision grip control, autistic individuals show elevated variability of force output that is associated with atypical activation and functional connectivity within parietal-cerebellar brain networks^81,82,99^. During precision gripping, autistic individuals also show reduced functional connectivity between the anterior cingulate and motor (M1) cortices, as well as increased activation within posterior parietal cortex that are associated with increased grip force variability^82^. These findings indicate that elevated grip force variability in ASD is associated with reduced modulation of the same sensorimotor, cognitive and attentional networks that are associated with elevated gait variability in other clinical populations, suggesting that functional differences in these networks likely contribute to elevated variability of multiple motor behaviors in ASD including gait and manual motor control.

### Clinical correlations.

We found that increased gait variability was moderately associated with clinical ratings of ritualistic behavior. This finding suggests that gait control differences may share important neurodevelopmental mechanisms with certain core autistic traits. While this correlation was marginally significant and should be interpreted with caution, it is consistent with findings from other studies demonstrating a relationship between motor variability and repetitive behaviors in autistic individuals^85,100^. Separate studies also have found associations between repetitive behavior and the structure and function of the cerebellum^99,101,102^. This includes a finding that repetitive behavior is associated with increased activation of cerebellar lobule VIIb during a precision gripping task for which autistic individuals show elevated motor variability^99^. Together these findings suggest that shared or overlapping cerebellar mechanisms may underlie both elevated motor variability and repetitive behavior in autistic individuals.

## LIMITATIONS

The present study used a composite metric of gait variability, the CGVS, which differentiated autistic from NT individuals and demonstrated that gait variability is elevated across ages in ASD relative to NT. This is a critical step towards developing more quantitative, objective measures of atypical sensorimotor function in ASD that could be used for tracking changes in sensorimotor differences and their underlying mechanisms across the lifespan. While the CGVS appears to show benefit relative to discrete gait variability metrics in differentiating autistic and NT individuals (as evidenced by the relatively large AUC in the ROC curve), the present study was not appropriately powered to statistically compare the sensitivity and specificity of the CGVS relative to the discrete gait variability metrics. Future studies using large samples are needed to evaluate the robustness of the CGVS relative to discrete gait metrics for distinguishing autistic from NT and non-autistic clinical populations.

Additionally, while the present study found persistently elevated gait variability in autistic individuals relative to NT individuals in a sample of individuals ages four to 35 years old, this is a cross-sectional study, and the 20–30 year age range is sparsely sampled. Future longitudinal studies of gait development across a broad age range are needed to determine developmental mechanisms leading to elevated gait variability across middle and later adulthood.

## CONCLUSIONS

Our findings document a new robust measure of gait variability in ASD that offers promise for understanding key mechanisms of sensorimotor differences in ASD across the lifespan. Understanding these sensorimotor differences is important for both determining neurodevelopmental processes associated with ASD, as well as establishing novel biomarkers that may be sensitive to important changes related to development, interventions, or neurological or psychiatric conditions that often co-occur with ASD (e.g., seizures). Our results also suggest that sensorimotor behaviors show elevated variability in autistic individuals across childhood and adulthood, implicating persistent neurodevelopmental differences in brain networks supporting modulation of intrinsic variability of motor behavior.

## Supplementary Material

Supplementary Files

This is a list of supplementary files associated with this preprint. Click to download.

- GA.png
- SupplementaryTables.docx

## Figures and Tables

**Table 1:** Demographic and clinical characteristics of autistic individuals (ASD) and neurotypical controls (NT)

Sample size (n)	ASD (mean ± SD)	NT (mean ± SD)	t/χ^2^	*p*
	60	53	–	–
Age (years)	15.700 ± 6.071	18.170 ± 8.274	−1.788	0.077
Sex (M/F)^a^	43/17	34/19	0.732	0.392
Height (cm)	161.735 ± 16.913	159.135 ± 18.590	0.778	0.438
Weight (kg)	61.434 ± 23.690	59.578 ± 25.826	0.399	0.691
Full-scale IQ	103.800 ± 14.907	109.615 ± 9.547	−2.490	**0.014** *
Verbal IQ	101.000 ± 16.239	108.692 ± 11.290	−2.940	**0.004** **
Performance IQ	106.100 ± 15.208	107.981 ± 10.584	−0.767	0.445
ADOS-2^b^	6.321 ± 1.869	–	–	–
RBS-R^c^	31.310 ± 19.395	2.865 ± 4.498	10.379	**<0.001** ***

a Chi-square statistical results

b ADOS-2 calibrated severity score

c RBS-R total raw score.

* p < 0.05

** p < 0.01

*** p < 0.001

**Table 2:** Descriptive statistics of gait standard deviation variables and MANCOVA results of autistic individuals (ASD) and neurotypical controls (NT)

Gait standard deviation measurement	ASD (mean ± sd)	NT (mean ± sd)	F	*p_raw_*	*p_FDR_*
Cadence.sd (steps/min)	6.612 ± 4.548	5.237 ± 3.879	5.628	**0.020**	**0.030** *
StepWidth.sd (cm)	2.615 ± 1.078	2.106 ± 0.769	8.017	**0.006**	**0.011** *
StepVelocity.sd (cm/s)	9.935 ± 4.706	7.587 ± 4.632	10.240	**0.002**	**0.009** **
HipAngle.HS.sd (deg)	2.173 ± 1.107	1.663 ± 0.788	8.551	**0.004**	**0.012** *
KneeAngle.HS.sd (deg)	2.062 ± 1.256	1.514 ± 0.867	8.484	**0.004**	**0.010** *
AnkleAngle.HS.sd (deg)	2.168 ± 1.196	1.684 ± 1.761	5.065	**0.027**	**0.035** *
HipAngle.TO.sd (deg)	2.439 ± 1.570	1.632 ± 0.781	13.208	**<0.001**	**0.004** **
KneeAngle.TO.sd (deg)	2.697 ± 1.541	2.375 ± 1.303	2.164	0.144	0.162
AnkleAngle.TO.sd (deg)	3.566 ± 2.391	3.184 ± 2.149	1.694	0.196	0.196

sd: standard deviation. HS: heel-strike; TO: toe-off.

* p < 0.05

** p < 0.01

*** p < 0.001

**Table 3:** Canonical correlation analysis results

Canonical variate	Canonical correlation ρ	Wilks’ Lambda	F	df_1_	df_2_	*p*
**1**	**0.662**	**0.475**	**2.820**	**28**	**343.950**	**<0.001** ***
2	0.286	0.845	0.929	18	272.014	0.543
3	0.274	0.920	0.829	10	194.000	0.601
4	0.077	0.994	0.145	4	98.000	0.965

* p < 0.05

** p < 0.01

*** p < 0.001

**Table 4:** ROC performance (AUC with 95% CI) of CGVS and discrete gait metrics for differentiating ASD from NT

Measure	AUC (95% CI)
CGVS	0.729 (0.629–0.828)
Cadence.sd (steps/min)	0.599 (0.491–0.706)
StepWidth.sd (cm)	0.650 (0.545–0.756)
StepVelocity.sd (cm/s)	0.679 (0.577–0.781)
HipAngle.HS.sd (deg)	0.648 (0.544–0.752)
KneeAngle.HS.sd (deg)	0.638 (0.533–0.743)
AnkleAngle.HS.sd (deg)	0.697 (0.592–0.802)
HipAngle.TO.sd (deg)	0.659 (0.555–0.762)

AUC = area under the ROC curve; CI = 95% confidence interval; ASD coded as the positive class (1); HS = heel strike; TO = toe-off

## Data Availability

All data are available from the corresponding authors upon reasonable request.

## References

[1] American Psychiatric Association (2022) Diagnostic and Statistical Manual of Mental Disorders. 5th, text revision ed

[2] Bhat AN (2021) Motor Impairment Increases in Children With Autism Spectrum Disorder as a Function of Social Communication, Cognitive and Functional Impairment, Repetitive Behavior Severity, and Comorbid Diagnoses: A SPARK Study Report. Autism Res 14(1):202–219. 10.1002/aur.2453 33300285 PMC8176850

[3] Green D, Charman T, Pickles A (2009) Impairment in movement skills of children with autistic spectrum disorders. Dev Med Child Neurol 51(4):311–316 19207298 10.1111/j.1469-8749.2008.03242.x

[4] Zhou B, Xu Q, Li H (2022) Motor impairments in Chinese toddlers with autism spectrum disorder and its relationship with social communicative skills. Front Psychiatry 13:938047. 10.3389/fpsyt.2022.938047 36311507 PMC9613953

[5] Licari MK, Alvares GA, Varcin K (2020) Prevalence of Motor Difficulties in Autism Spectrum Disorder: Analysis of a Population-Based Cohort. Autism Res 13(2):298–306. 10.1002/aur.2230 31625694

[6] Landa RJ, Haworth JL, Nebel MB, Ready(2016) Set, Go! Low Anticipatory Response during a Dyadic Task in Infants at High Familial Risk for Autism. Front Psychol 7. 10.3389/fpsyg.2016.00721 PMC4879330 27252667

[7] Wilson RB, Burdekin ED, Jackson NJ (2024) Slower pace in early walking onset is related to communication, motor skills, and adaptive function in autistic toddlers. Autism Res 17(1):27–36. 10.1002/aur.3067 38009228 PMC10842796

[8] Ravizza SM, Solomon M, Ivry RB, Carter CS (2013) Restricted and repetitive behaviors in autism spectrum disorders: The relationship of attention and motor deficits. Dev Psychopathol 25(03):773–784. 10.1017/S0954579413000163 23880391 PMC5538881

[9] Hedgecock JB, Dannemiller LA, Shui AM, Rapport MJ, Katz T (2018) Associations of Gross Motor Delay, Behavior, and Quality of Life in Young Children With Autism Spectrum Disorder. Phys Ther 98(4):251–259. 10.1093/ptj/pzy006 29325143

[10] LeBarton ES, Landa RJ (2019) Infant motor skill predicts later expressive language and autism spectrum disorder diagnosis. Infant Behav Dev 54:37–47. 10.1016/j.infbeh.2018.11.003 30557704

[11] Sacrey LAR, Zwaigenbaum L, Bryson S, Brian J, Smith IM (2018) The reach-to-grasp movement in infants later diagnosed with autism spectrum disorder: a high-risk sibling cohort study. J Neurodev Disord 10(1):41. 10.1186/s11689-018-9259-4 30587102 PMC6307213

[12] Leezenbaum NB, Iverson JM (2019) Trajectories of Posture Development in Infants With and Without Familial Risk for Autism Spectrum Disorder. J Autism Dev Disord 49(8):3257–3277. 10.1007/s10803-019-04048-3 31079276

[13] Unruh KE, McKinney WS, Bojanek EK, Fleming KK, Sweeney JA, Mosconi MW (2021) Initial action output and feedback-guided motor behaviors in autism spectrum disorder. Mol Autism 12(1):52. 10.1186/s13229-021-00452-8 34246292 PMC8272343

[14] Manicolo O, Brotzmann M, Hagmann-von Arx P, Grob A, Weber P (2019) Gait in children with infantile/atypical autism: Age-dependent decrease in gait variability and associations with motor skills. Eur J Pediatr Neurol 23(1):117–125. 10.1016/j.ejpn.2018.09.011 30482681

[15] Fears NE, Sherrod GMC, Templin TN, Bugnariu NL, Patterson RM, Miller HL (2023) Community-based postural control assessment in autistic individuals indicates a similar but delayed trajectory compared to neurotypical individuals. Autism Res 16(3):543–557. 10.1002/aur.2889 36627838 PMC10023334

[16] Alsaedi RH (2020) An Assessment of the Motor Performance Skills of Children with Autism Spectrum Disorder in the Gulf Region. Brain Sci 10(9):607. 10.3390/brainsci10090607 32899306 PMC7564795

[17] Linke AC, Kinnear MK, Kohli JS (2020) Impaired motor skills and atypical functional connectivity of the sensorimotor system in 40- to 65-year-old adults with autism spectrum disorders. Neurobiol Aging 85:104–112. 10.1016/j.neurobiolaging.2019.09.018 31732217 PMC6948185

[18] Sparaci L, Northrup JB, Capirci O, Iverson JM (2018) From Using Tools to Using Language in Infant Siblings of Children with Autism. J Autism Dev Disord 48(7):2319–2334. 10.1007/s10803-018-3477-1 29429008 PMC6592270

[19] Grace N, Johnson BP, Rinehart NJ, Enticott PG (2018) Are Motor Control and Regulation Problems Part of the ASD Motor Profile? A Handwriting Study. Dev Neuropsychol 43(7):581–594. 10.1080/87565641.2018.1504948 30124332

[20] Jequier Gygax M, Maillard AM, Favre J (2021) Could Gait Biomechanics Become a Marker of Atypical Neuronal Circuitry in Human Development?—The Example of Autism Spectrum Disorder. Front Bioeng Biotechnol 9. 10.3389/fbioe.2021.624522 PMC8009281 33796508

[21] Adolph KE, Tamis-LeMonda CS (2014) The Costs and Benefits of Development: The Transition From Crawling to Walking. Child Dev Perspect 8(4):187–192. 10.1111/cdep.12085 25774213 PMC4357016

[22] Thurman SL, Corbetta D (2019) Changes in Posture and Interactive Behaviors as Infants Progress From Sitting to Walking: A Longitudinal Study. Front Psychol 10:822. 10.3389/fpsyg.2019.00822 31031682 PMC6473077

[23] Malloggi C, Rota V, Catino L (2019) Three-dimensional path of the body centre of mass during walking in children: an index of neural maturation. Int J Rehabil Res 42(2):112–119. 10.1097/MRR.0000000000000345 30882528 PMC6493692

[24] Voss S, Joyce J, Biskis A (2020) Normative database of spatiotemporal gait parameters using inertial sensors in typically developing children and young adults. Gait Posture 80:206–213. 10.1016/j.gaitpost.2020.05.010 32531757 PMC7388584

[25] Kraan CM, Tan AHJ, Cornish KM (2017) The developmental dynamics of gait maturation with a focus on spatiotemporal measures. Gait Posture 51:208–217. 10.1016/j.gaitpost.2016.10.021 27816899

[26] Alderson LM, Joksaite SX, Kemp J (2019) Age-related gait standards for healthy children and young people: the GOS-ICH paediatric gait centiles. Arch Dis Child 104(8):755–760. 10.1136/archdischild-2018-316311 30910816

[27] Kung SM, Fink PW, Legg SJ, Ali A, Shultz SP (2019) Age-dependent variability in spatiotemporal gait parameters and the walk-to-run transition. Hum Mov Sci 66:600–606. 10.1016/j.humov.2019.06.012 31277034

[28] Hausdorff JM, Zemany L, Peng CK, Goldberger AL (1999) Maturation of gait dynamics: stride-to-stride variability and its temporal organization in children. J Appl Physiol 86(3):1040–1047 10066721 10.1152/jappl.1999.86.3.1040

[29] Price R, Choy NL (2019) Investigating the Relationship of the Functional Gait Assessment to Spatiotemporal Parameters of Gait and Quality of Life in Individuals With Stroke. J Geriatr Phys Ther 42(4):256–264. 10.1519/JPT.0000000000000173 29324509

[30] Larsson J, Hansson W, Israelsson Larsen H, Koskinen LOD, Eklund A, Malm J (2025) Higher-level gait disorders: a population-based study on prevalence, quality of life, depression and confidence in gait and balance. BMJ Neurol Open 7(1):e000992. 10.1136/bmjno-2024-000992 PMC11907029 40092839

[31] Marincolo JCS, de Assumpção D, Santimaria MR (2024) Low grip strength and gait speed as markers of dependence regarding basic activities of daily living: the FIBRA study. Einstein (Sao Paulo) 22:eAO0637. 10.31744/einstein_journal/2024AO0637 38808796 PMC11155723

[32] Park J, Kim TH (2019) The effects of balance and gait function on quality of life of stroke patients. NeuroRehabilitation 44(1):37–41. 10.3233/NRE-182467 30741699

[33] Weiss MJ, Moran MF, Parker ME, Foley JT (2013) Gait analysis of teenagers and young adults diagnosed with autism and severe verbal communication disorders. Front Integr Neurosci 7. 10.3389/fnint.2013.00033 PMC3657630 23730274

[34] Ganai UJ, Ratne A, Bhushan B, Venkatesh KS (2025) Early detection of autism spectrum disorder: gait deviations and machine learning. Sci Rep 15(1):873. 10.1038/s41598-025-85348-w 39757284 PMC11701103

[35] Nobile M, Perego P, Piccinini L (2011) Further evidence of complex motor dysfunction in drug naïve children with autism using automatic motion analysis of gait. Autism 15(3):263–283. 10.1177/1362361309356929 21478224

[36] Cho AB, Otte K, Baskow I (2022) Motor signature of autism spectrum disorder in adults without intellectual impairment. Sci Rep 12(1):7670. 10.1038/s41598-022-10760-5 35538115 PMC9090847

[37] Wu X, Dickin DC, Bassette L, Ashton C, Wang H (2024) Clinical gait analysis in older children with autism spectrum disorder. Sports Med Health Sci 6(2):154–158. 10.1016/j.smhs.2023.10.007 38708319 PMC11067783

[38] Calhoun M, Longworth M, Chester VL (2011) Gait patterns in children with autism. Clin Biomech (Bristol) 26(2):200–206. 10.1016/j.clinbiomech.2010.09.013 20934239

[39] Dufek JS, Eggleston JD, Harry JR, Hickman RA (2017) A Comparative Evaluation of Gait between Children with Autism and Typically Developing Matched Controls. Med Sci 5(1):1. 10.3390/medsci5010001 PMC5635776 29099017

[40] Lum JAG, Shandley K, Albein-Urios N (2021) Meta-Analysis Reveals Gait Anomalies in Autism. Autism Res 14(4):733–747. 10.1002/aur.2443 33289353

[41] Li Y, Koldenhoven RM, Liu T, Venuti CE (2021) Age-related gait development in children with autism spectrum disorder. Gait Posture 84:260–266. 10.1016/j.gaitpost.2020.12.022 33383537

[42] Bennett HJ, Jones T, Valenzuela KA, Haegele JA (2021) Inter and intra-limb coordination variability during walking in adolescents with autism spectrum disorder. Clin Biomech (Bristol) 89:105474. 10.1016/j.clinbiomech.2021.105474 34500337

[43] Hausdorff JM (2007) Gait dynamics, fractals and falls: Finding meaning in the stride-to-stride fluctuations of human walking. Hum Mov Sci 26(4):555–589. 10.1016/j.humov.2007.05.003 17618701 PMC2267927

[44] Kilby MC, Molenaar PCM, Newell KM (2015) Models of Postural Control: Shared Variance in Joint and COM Motions. PLoS ONE 10(5):e0126379. 10.1371/journal.pone.0126379 25973896 PMC4431684

[45] Kilby MC, Molenaar PC, Slobounov M, Newell SM (2017) Real-time visual feedback of COM and COP motion properties differentially modifies postural control structures. Exp Brain Res 235(1):109–120. 10.1007/s00221-016-4769-3 27644409

[46] Hotelling H (1936) Relations Between Two Sets of Variates. Biometrika 28(3/4):321–377. 10.2307/2333955

[47] Wu Y, Zhong Z, Lu M, He J (2011) Statistical analysis of gait maturation in children based on probability density functions. Annu Int Conf IEEE Eng Med Biol Soc 2011:1652–1655. 10.1109/IEMBS.2011.6090476 22254641

[48] Lasko-McCarthey P, Beuter A, Biden E (1990) Kinematic variability and relationships characterizing the development of walking. Dev Psychobiol 23(8):809–837. 10.1002/dev.420230805 2081577

[49] Rygelová M, Uchytil J, Torres IE, Janura M (2023) Comparison of spatiotemporal gait parameters and their variability in typically developing children aged 2, 3, and 6 years. PLoS ONE 18(5):e0285558. 10.1371/journal.pone.0285558 37167236 PMC10174554

[50] Unruh KE, McKinney WS, Bojanek EK, Fleming KK, Sweeney JA, Mosconi MW (2021) Initial action output and feedback-guided motor behaviors in autism spectrum disorder. Mol Autism 12(1):52. 10.1186/s13229-021-00452-8 34246292 PMC8272343

[51] Shafer RL, Wang Z, Bartolotti J, Mosconi MW (2021) Visual and somatosensory feedback mechanisms of precision manual motor control in autism spectrum disorder. J Neurodev Disord 13(1):32. 10.1186/s11689-021-09381-2 34496766 PMC8427856

[52] American Psychiatric Association (2013) Diagnostic and Statistical Manual of Mental Disorders (DSM-5^®^). American Psychiatric Publishing

[53] Rutter M, Bailey A, Lord C (2003) The Social Communication Questionnaire: Manual. Western Psychological Services

[54] Baron-Cohen S, Wheelwright S, Skinner R, Martin J, Clubley E (2001) The Autism-Spectrum Quotient (AQ): Evidence from Asperger Syndrome/High-Functioning Autism, Males and Females, Scientists and Mathematicians. J Autism Dev Disord 31(1):13 10.1023/a:1005653411471 11439754

[55] Constantino JN, Todd RD (2005) Intergenerational transmission of subthreshold autistic traits in the general population. Biol Psychiatry 57(6):655–660. 10.1016/j.biopsych.2004.12.014 15780853

[56] Constantino JN, Gruber CP (2012) The Social Responsiveness Scale Manual (SRS-2). Second. Western Psychological Services

[57] Lord C, Rutter M, DiLavore PC, Risi S, Gotham K, Bishop S (2012) Autism Diagnostic Observation Schedule: ADOS-2. Western Psychological Services

[58] Lord C, Rutter M, Le Couteur A (1994) Autism Diagnostic Interview-Revised: a revised version of a diagnostic interview for caregivers of individuals with possible pervasive developmental disorders. J Autism Dev Disord 24(5):659–685 7814313 10.1007/BF02172145

[59] Bodfish JW, Symons FJ, Parker DE, Lewis MH (2000) Varieties of repetitive behavior in autism: Comparisons to mental retardation. J Autism Dev Disord 30(3):237–243 11055459 10.1023/a:1005596502855

[60] Wechsler D, Zhou X (2011) WASI-II: Wechsler Abbreviated Scale of Intelligence. Second. The Psychological Corporation

[61] Hus V, Lord C (2014) The Autism Diagnostic Observation Schedule, Module 4: Revised Algorithm and Standardized Severity Scores. J Autism Dev Disord 44(8):1996–2012. 10.1007/s10803-014-2080-3 24590409 PMC4104252

[62] Gotham K, Pickles A, Lord C (2009) Standardizing ADOS Scores for a Measure of Severity in Autism Spectrum Disorders. J Autism Dev Disord 39(5):693–705. 10.1007/s10803-008-0674-3 19082876 PMC2922918

[63] Scataglini S, Dellaert L, Meeuwssen L, Staeljanssens E, Truijen S (2025) The difference in gait pattern between adults with obesity and adults with a normal weight, assessed with 3D-4D gait analysis devices: a systematic review and meta-analysis. Int J Obes (Lond) 49(4):541–553. 10.1038/s41366-024-01659-4 39562690

[64] Thevenon A, Gabrielli F, Lepvrier J (2015) Collection of normative data for spatial and temporal gait parameters in a sample of French children aged between 6 and 12. Annals Phys Rehabilitation Med 58(3):139–144. 10.1016/j.rehab.2015.04.001 25952820

[65] Frimenko R, Goodyear C, Bruening D (2015) Interactions of sex and aging on spatiotemporal metrics in non-pathological gait: a descriptive meta-analysis. Physiotherapy 101(3):266–272. 10.1016/j.physio.2015.01.003 25702092

[66] Benjamini Y, Hochberg Y (1995) Controlling the False Discovery Rate: A Practical and Powerful Approach to Multiple Testing. J Roy Stat Soc: Ser B (Methodol) 57(1):289–300. 10.1111/j.2517-6161.1995.tb02031.x

[67] Hanley JA, McNeil BJ (1982) The meaning and use of the area under a receiver operating characteristic (ROC) curve. Radiology 143(1):29–36. 10.1148/radiology.143.1.7063747 7063747

[68] Fisher RA (1921) On the probable error of a coefficient of correlation deduced from a small sample. Metron 1:3–21

[69] Steiger JH (1980) Tests for comparing elements of a correlation matrix. Psychol Bull 87:245–251. 10.1037/0033-2909.87.2.245

[70] Gouelle A, Mégrot F (2016) Interpreting Spatiotemporal Parameters, Symmetry, and Variability in Clinical Gait Analysis. Handbook of Human Motion. Springer, Cham, pp 1–20. doi:10.1007/978-3-319-30808-1_35-1

[71] Applequist BC, Motz ZL, Kyvelidou A (2023) Spatiotemporal Gait Variability in Children Aged 2 to 10 Decreases throughout Pre-Adolescence. Biomechanics 3(4):571–582. 10.3390/biomechanics3040046

[72] Ilg W, Golla H, Thier P, Giese MA (2007) Specific influences of cerebellar dysfunctions on gait. Brain 130(Pt 3):786–798. 10.1093/brain/awl376 17287287

[73] Sigurdsson HP, Yarnall AJ, Galna B (2022) Gait-Related Metabolic Covariance Networks at Rest in Parkinson’s Disease. Mov Disord 37(6):1222–1234. 10.1002/mds.28977 35285068 PMC9314598

[74] Wuehr M, Schniepp R, Schlick C (2014) Sensory loss and walking speed related factors for gait alterations in patients with peripheral neuropathy. Gait Posture 39(3):852–858. 10.1016/j.gaitpost.2013.11.013 24342450

[75] Byun S, Lee HJ, Kim JS (2023) Exploring shared neural substrates underlying cognition and gait variability in adults without dementia. Alzheimers Res Ther 15(1):206. 10.1186/s13195-023-01354-y 38012628 PMC10680297

[76] Fernandez NB, Hars M, Trombetti A, Vuilleumier P (2019) Age-related changes in attention control and their relationship with gait performance in older adults with high risk of falls. NeuroImage 189:551–559. 10.1016/j.neuroimage.2019.01.030 30660655

[77] Wuehr M, Schniepp R, Schlick C (2014) Sensory loss and walking speed related factors for gait alterations in patients with peripheral neuropathy. Gait Posture 39(3):852–858. 10.1016/j.gaitpost.2013.11.013 24342450

[78] Schmitt LM, Cook EH, Sweeney JA, Mosconi MW (2014) Saccadic eye movement abnormalities in autism spectrum disorder indicate dysfunctions in cerebellum and brainstem. Mol Autism 5(1):47. 10.1186/2040-2392-5-47 25400899 PMC4233053

[79] Zalla T, Seassau M, Cazalis F, Gras D, Leboyer M (2018) Saccadic eye movements in adults with high-functioning autism spectrum disorder. Autism 22(2):195–204. 10.1177/1362361316667057 29490485

[80] Mosconi MW, Mohanty S, Greene RK, Cook EH, Vaillancourt DE, Sweeney JA (2015) Feedforward and Feedback Motor Control Abnormalities Implicate Cerebellar Dysfunctions in Autism Spectrum Disorder. J Neurosci 35(5):2015–2025. 10.1523/JNEUROSCI.2731-14.2015 25653359 PMC4315832

[81] Lepping RJ, McKinney WS, Magnon GC (2021) Visuomotor brain network activation and functional connectivity among individuals with autism spectrum disorder. Hum Brain Mapp Published online Oct 30. 10.1002/hbm.25692 PMC8720186 34716740

[82] Unruh KE, Bartolotti JV, McKinney WS, Schmitt LM, Sweeney JA, Mosconi MW (2023) Functional connectivity of cortical-cerebellar networks in relation to sensorimotor behavior and clinical features in autism spectrum disorder. Cereb Cortex 33(14):8990–9002. 10.1093/cercor/bhad177 37246152 PMC10350826

[83] Cherylm Glazebrook, Gonzalez D, Hansen, Elliott D (2009) The role of vision for online control of manual aiming movements in persons with autism spectrum disorders. Autism 13(4):411–433. 10.1177/1362361309105659 19535469

[84] Lim YH, Lee HC, Falkmer T (2018) Effect of Visual Information on Postural Control in Adults with Autism Spectrum Disorder. J Autism Dev Disord 72:175–181. 10.1007/s10803-018-3634-6 29882108

[85] Wang Z, Hallac RR, Conroy KC (2016) Postural orientation and equilibrium processes associated with increased postural sway in autism spectrum disorder (ASD). J Neurodev Disord 8:43. 10.1186/s11689-016-9178-1 27933108 PMC5124312

[86] Rakié C, Iverson JM, Bailes AH, Richard JJ, Eack SM, Redfern MS (2021) Attention and sensory integration for postural control in young adults with autism spectrum disorders. Exp Brain Res 239(5):1417–1426. 10.1007/s00221-021-06058-z 33675379

[87] Bick NA, Redfern MS, Jennings JR, Eack SM, Iverson JM, Cham R (2024) Attention and sensory integration for gait in young adults with autism spectrum disorder. Gait Posture 112:74–80. 10.1016/j.gaitpost.2024.04.035 38749292 PMC11193611

[88] Eggleston JD, Harry JR, Cereceres PA (2020) Lesser Magnitudes of Lower Extremity Variability during Terminal Swing Characterizes Walking Patterns in Children with Autism. Clin Biomech (Bristol Avon) 76:105031. 10.1016/j.clinbiomech.2020.105031 PMC7282997 32408186

[89] Lo OY, Halko MA, Zhou J, Harrison R, Lipsitz LA, Manor B (2017) Gait Speed and Gait Variability Are Associated with Different Functional Brain Networks. Front Aging Neurosci 9:390. 10.3389/fnagi.2017.00390 29249961 PMC5715372

[90] Hausdorff JM (2009) Gait dynamics in Parkinson’s disease: common and distinct behavior among stride length, gait variability, and fractal-like scaling. Chaos 19(2):026113. 10.1063/1.3147408 19566273 PMC2719464

[91] Bertuccelli M, Bisiacchi P, Del Felice A (2024) Disentangling Cerebellar and Parietal Contributions to Gait and Body Schema: A Repetitive Transcranial Magnetic Stimulation Study. Cerebellum 23(5):1848–1858. 10.1007/s12311-024-01678-x 38438828 PMC11489286

[92] Ilg W, Christensen A, Mueller OM, Goericke SL, Giese MA, Timmann D (2013) Effects of cerebellar lesions on working memory interacting with motor tasks of different complexities. J Neurophysiol 110(10):2337–2349. 10.1152/jn.00062.2013 23966680

[93] Lo OY, Halko MA, Devaney KJ, Wayne PM, Lipsitz LA, Manor B (2021) Gait Variability Is Associated With the Strength of Functional Connectivity Between the Default and Dorsal Attention Brain Networks: Evidence From Multiple Cohorts. J Gerontol Biol Sci Med Sci 76(10):e328–e334. 10.1093/gerona/glab200 PMC8436983 34244725

[94] Nishida A, Shima A, Kambe D (2024) Frontoparietal-Striatal Network and Nucleus Basalis Modulation in Patients With Parkinson Disease and Gait Disturbance. Neurology 103(3):e209606. 10.1212/WNL.0000000000209606 38976821

[95] Wang Y, Yu N, Lu J (2023) Increased Effective Connectivity of the Left Parietal Lobe During Walking Tasks in Parkinson’s Disease. J Parkinsons Dis 13(2):165–178. 10.3233/JPD-223564 36872789 PMC10041419

[96] Khan AJ, Nair A, Keown CL, Datko MC, Lincoln AJ, Müller RA (2015) Cerebro-cerebellar Resting-State Functional Connectivity in Children and Adolescents with Autism Spectrum Disorder. Biol Psychiatry 78(9):625–634. 10.1016/j.biopsych.2015.03.024 25959247 PMC5708535

[97] Oldehinkel M, Mennes M, Marquand A (2018) Altered Connectivity Between Cerebellum, Visual, and Sensory-Motor Networks in Autism Spectrum Disorder: Results from the EU-AIMS Longitudinal European Autism Project. Biol Psychiatry: Cogn Neurosci Neuroimaging 4(3):260–270. 10.1016/j.bpsc.2018.11.010 30711508

[98] Wang Z, Wang Y, Sweeney JA, Gong Q, Lui S, Mosconi MW (2019) Resting-State Brain Network Dysfunctions Associated With Visuomotor Impairments in Autism Spectrum Disorder. Front Integr Neurosci 13. 10.3389/fnint.2019.00017 PMC6554427 31213995

[99] Unruh KE, Martin LE, Magnon G, Vaillancourt DE, Sweeney JA, Mosconi MW (2019) Cortical and subcortical alterations associated with precision visuomotor behavior in individuals with autism spectrum disorder. J Neurophysiol 122(4):1330–1341. 10.1152/jn.00286.2019 31314644 PMC6843107

[100] Bojanek EK, Wang Z, White SP, Mosconi MW (2020) Postural control processes during standing and step initiation in autism spectrum disorder. J Neurodev Disord 12(1):1. 10.1186/s11689-019-9305-x 31906846 PMC6945692

[101] Wolff JJ, Swanson MR, Elison JT (2017) Neural circuitry at age 6 months associated with later repetitive behavior and sensory responsiveness in autism. Mol Autism 8(1):8. 10.1186/s13229-017-0126-z 28316772 PMC5351210

[102] McKinney WS, Kelly SE, Unruh KE (2022) Cerebellar Volumes and Sensorimotor Behavior in Autism Spectrum Disorder. Front Integr Neurosci 16:821109. 10.3389/fnint.2022.821109 35592866 PMC9113114

[103] Feaster DJ, Mikulich-Gilbertson S, Brincks AM (2011) Modeling site effects in the design and analysis of multi-site trials. Am J Drug Alcohol Abuse 37(5):383–391. 10.3109/00952990.2011.600386 21854281 PMC3281513

